# Packaging and Delivery of Asthma Therapeutics

**DOI:** 10.3390/pharmaceutics14010092

**Published:** 2021-12-31

**Authors:** Bryan J. Mathis, Misa Kusumoto, Alexander Zaboronok, Yuji Hiramatsu

**Affiliations:** 1 International Medical Center, University of Tsukuba Hospital, Tsukuba 305-8576, Japan; yuji3@md.tsukuba.ac.jp; 2 School of Medicine, University of Tsukuba, Tsukuba 305-8575, Japan; misa.kusumoto126@gmail.com; 3 Department of Neurosurgery, Faculty of Medicine, University of Tsukuba, Tsukuba 305-8575, Japan; a.zaboronok@md.tsukuba.ac.jp

**Keywords:** asthma, nanoparticle, exosome, liposome, inhaled medications, drug packaging

## Abstract

Asthma is a life-altering, chronic disease of heterogenous origin that features a complex interplay of immune and environmental signaling. Although very little progress has been made in prevention, diverse types of medications and delivery systems, including nanoscale systems, have been or are currently being developed to control airway inflammation and prevent exacerbations and fibrosis. These medications are delivered through mechanical methods, with various inhalers (with benefits and drawbacks) existing, and new types offering some variety in delivery. Of particular interest is the progress being made in nanosized materials for efficient penetration into the epithelial mucus layer and delivery into the deepest parts of the lungs. Liposomes, nanoparticles, and extracellular vesicles, both natural and synthetic, have been explored in animal models of asthma and have produced promising results. This review will summarize and synthesize the latest developments in both macro-(inhaler) and micro-sized delivery systems for the purpose of treating asthma patients.

## 1. Introduction

Asthma is a chronic disease featuring immune dysregulation as its core pathology. It affects hundreds of millions globally, evidenced by diagnosis rates that are climbing yearly, even as mortality decreases [1]. Hallmark symptoms of this heterogenous condition include lowered forced expiratory volume, chronic cough, airway constriction, dyspnea, hypoxia, and wheezing [2]. However, while medical science has been unable to fully elucidate the detailed mechanisms of asthma pathology to develop preventative measures, it continues to research new therapies to prevent exacerbations and mortality. These therapies block immune signal transduction upon the challenge of airway epithelial cells, with allergic signals and numerous pathways being extensively reviewed elsewhere [2,3,4]. In order to fulfill their intended use, drugs need the correct molecular packaging as well as effective mechanical delivery systems to provide rapid and sustained relief from asthma. What follows is a unique, all-aspect, literature-based survey of current and promising pharmaceutics for asthma, traditional and mechanical delivery systems, and the newest nano-molecular packaging strategies. 

### Asthma Pathology

**Figure 1:**
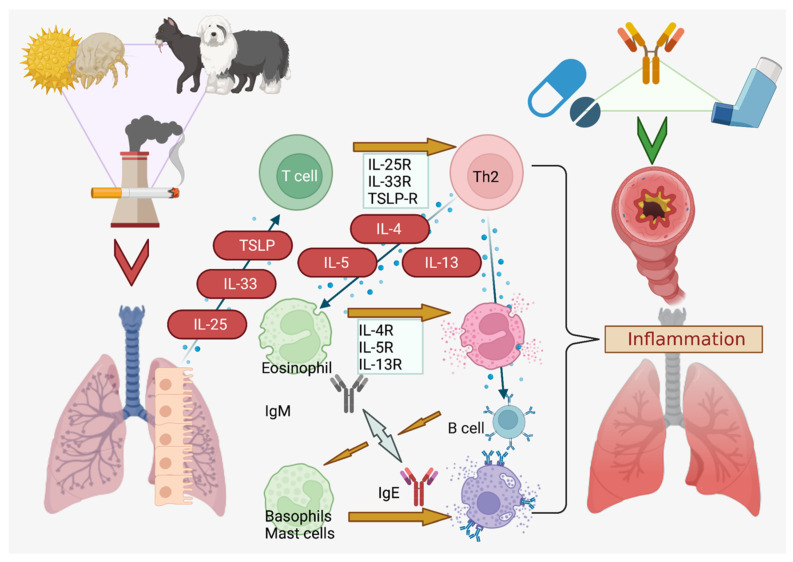
Sources and Pathway of Asthmatic Exacerbations. Dust, pollen, pet dander, and smoke from cigarettes or other sources are potent asthmatic triggers [16,17]. Created in BioRender.com.

Asthma is a type 2 immune disorder that is classified phenotypically, with eosinophilic, neutrophilic, mixed granulocytic, and paucigranulocytic subtypes that are distinguished by sputum analysis for immune presence [5]. Three chief phenotypes are currently recognized: inflammation mediated by eosinophils (late-onset, early-onset), obesity or smoking-associated (lifestyle), and allergic hypersensitivity in the trachea or bronchial airways [5]. Remodeling of the respiratory tract, with excessive fibrosis and scarring from collagen deposition, occurs due to chronic inflammation, and this process is irreversible [6]. It is thus imperative to rapidly reduce inflammation within the airways as well as prevent errant activation of the allergen hypersensitivity mechanism. Genetic components play a role, with protective transforming growth factor-β (TGF-*β*) signaling mutations having been reported, but, in general, avoidance of pet dander, plant pollens, dust, pm2.5 pollution, ozone, and cold air are also required to minimize reactive airway hypersensitivity (Figure 1) [7,8].

Asthma can also be classified by the age of onset into childhood or adult-onset asthma. Childhood asthma, mainly related to atopy and viral infection below the age of 6 (resulting in a decrease of interferon-β and interferon-γ in the bronchial cells), is more common in boys than in girls, due to enhanced allergic reaction and higher IgE levels [9,10,11]. On the other hand, adult-onset asthma is linked to negative lifestyle factors, such as smoking, pollution, obesity, GERD, AERD, and occupation [12]. Women tend to have worse symptoms and severe asthma attacks than men, due to female sex hormones and a smaller airway diameter than men [11,13,14,15]. 

Briefly, mast cell degranulation, interleukin (IL)-4, IL-5 and IL-13 are downstream mediators of asthmatic exacerbations, while CD4+T helper-2 (Th2) cells play a central role in attracting eosinophils that release IL-5 to upregulate IL-4 and IL-13 [17]. B lymphocytes migrate via CCL and CXCL-motif chemokines (chiefly CCL21 and CXCL12/13) and are signaled by IL-4, IL-13, BLIMP1, and Xbox protein 1 to generate large amounts of IgE that stimulates the IgE receptor (FcεR1) on migrated mucosal mast cells, triggering a cascade comprised of cytokines, neutrophils, histamine, leukotrienes (Cys-LT), and prostaglandin D2 (PG-D2) (among others) [18,19,20]. Additionally, tryptase and chymase are released from mast cells that damage the airway cells with proteolytic action, while the fibrotic response is triggered by chymase activity on Smad, activating TGF-β [17,21]. This remodeling also activates angiogenesis and the release of fluid into the lumen, swelling the airway and constricting the breath [22]. The stiffness of collagen I, III, and IV that are deposited during airway remodeling permanently narrows the airway in concert with fibrosis [6,23]. 

## 2. Asthma Drug Metabolism (General)

### 2.1. “First-Pass” Cytochrome Metabolic System

Cytochrome P450 plays an important role in the human metabolism of medication by catalyzing oxidation of drugs after exposure to stomach acid in “first-pass” metabolism. Named after its absorption of 450nm light, this enzyme both activates and detoxifies chemicals [24]. In humans, 57 CYP cytochrome genes are known to carry out diverse metabolic functions, such as the role of microsomal P450s in mitochondrial steroid metabolism [24]. There are two main types of cytochromes: one that primarily detoxifies (located in the liver) and one that biosynthesizes endogenous compounds [25]. The basic mode of detoxifying cytochrome action is the iron-catalyzed oxidation of substrates using NADPH and oxygen to generate hydroxylated product and free radicals, facilitated by structures that freely bind to diverse substrates [26,27]. Drugs, such as asthma or other treatments intended for systemic diffusion through the blood, must therefore be either 1) resistant to catalytic action by P450 or 2) the metabolites must be the active form of the drug. 

Rajman et al. and Esteves et al. recently reported how ethnicity potentially affects cytochrome types as CYP2B6*6, CYP2C8*2, CYP2D6*3, CYP2D6*17, CYP2D6*29, CYP3A5*6, and CYP3A5*7 variability were related to the genetic diversity of African populations [28,29]. Several reports have also claimed that genetic polymorphisms of cytochromes are found to be related to certain disease and drug reactions [30]. Demographics additionally affect the activity of the CYP gene family, as CYP1A2, CYP2A6, CYP2B6, CYP2C8, CYP2C9, and CYP3A4/5 are known to have higher activity in older adults, while allelic variations may correlate with increased susceptibility to cancers (breast, lung, prostate, etc.) or nicotine (CYP2A6*2 1799T > A) metabolism [31]. As these important enzymes constitute the foundational processing for all substances entering the body, first-pass metabolism (in addition to age, gender, ethnic, and environmental variables) may limit the effectiveness of systemic oral medications for asthma control. 

### 2.2. Drug Metabolism Phases

**Figure 2:**
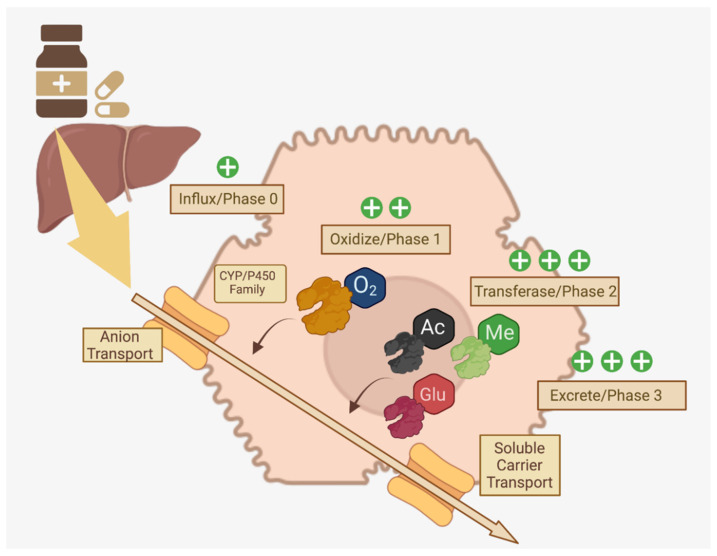
Multi-Phase Drug Metabolism in Hepatocytes. Distinct phases of drug metabolism that increase polarity through enzymes that modify xenobiotics via addition of functional groups are crucial for excretion of ingested medications [25,28,29,32]. Created in BioRender.com.

Membrane-bound drug transport proteins, such as organic anion/cation transporters, are crucial for the cellular influx of xenobiotics (often called “Phase 0”) into the proximity of Phase I/II enzymes and genetic polymorphisms in these transport proteins may be responsible for observed variability in asthma drug trials [32]. In Phase I, oxidation, reduction, and reactions to make drugs, vitamins, or xenobiotics more polar and hydrophilic are accomplished by CYP1, CYP2 and CYP3, featuring over 400 allelic variants [25,28]. In Phase II, diverse transferases (glutathione, glucuronyl, sulfur, acetyl, etc.) conjugate Phase I metabolites with functional groups to detoxify through solubilization to facilitate excretion [25,32]. Phase III primarily consists of excretion of these now-polar metabolites in the urine or feces after export by ATP binding cassette or solute carrier transporters [29]. A summary of this pathway is available in Figure 2.

## 3. Asthma Treatments Currently Available

The autoimmune/allergic component of asthma makes it a multifactorial chronic condition that is difficult to treat and seemingly impossible to cure. While past treatments attempted to counter asthmatic paroxysms by opening airways through alkaloid bronchodilators that had anti-cholinergic properties, recent biologic therapies focus on short-circuiting the transduction of specific immune products or signal transducers such as interleukins, prostaglandins, and inflammatory transcription factors [33]. To this end, diverse classes of therapeutics, each targeting a specific link in the inflammatory pathway, have been developed to offer rapid relief from airway constriction as well as prevent further exacerbations. Although inhaled medications are the gold standard for asthma, oral adjunct therapies may also be used for systemic control. Typically, these oral medications are packaged as pH-stable, crystalline salts to both extend shelf life and increase solubility in the acidic stomach environment [34]. These may be systemic regulators of inflammation (montelukast or pranlukast) or methylxanthines (theophylline) that inhibit phosphodiesterase (Table 1) [35]. However, the use of injectable, monoclonal antibody-derived drugs tailored for specific inhibition of interleukin-dominated inflammation has recently increased following successful clinical trials [16]. This section will briefly review the standard treatments as well as offer some insight on new classes of antibody-derived therapeutics that specifically target T lymphocyte and mast cell-mediated asthmatic inflammation. 

### 3.1. Steroids

**Figure 3:**
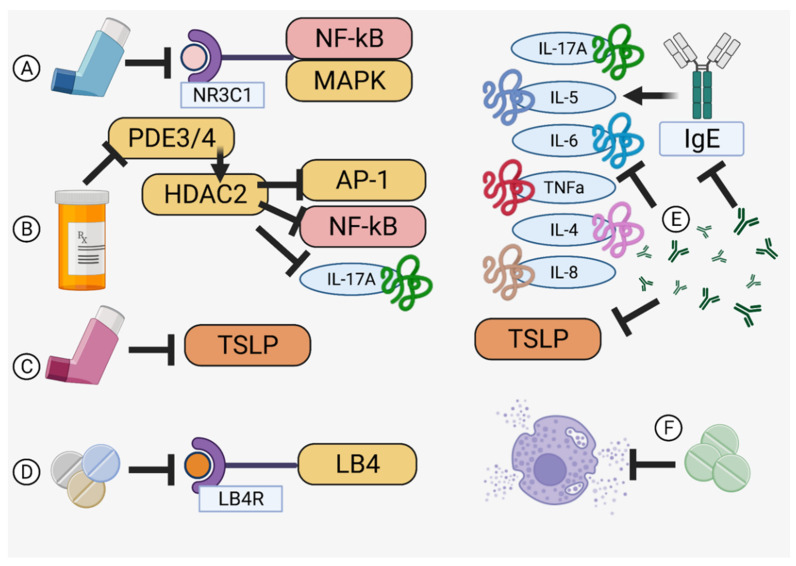
Modes of Action. Specificity for checkpoints in the asthma pathway is the hallmark of an effective therapy. (**A**) Inhaled corticosteroids [47,48], (**B**) theophylline [51,52], (**C**) long-acting beta agonists [57], (**D**) leukotriene receptor antagonists [62], (**E**) monoclonal antibodies [66,67,68], and (**F**) anti-histamines [71,72] offer diverse targeting specific to halting airway inflammation. Created in BioRender.com.

Inhaled corticosteroids (ICS), similar in function and structure to the cortisol produced by the adrenal glands, are a mainstay treatment for asthma that relieve and prevent asthmatic symptoms by suppressing eosinophils, T lymphocytes, mast cells, and dendritic cells (Table 1) [45,46]. Specific binding to the glucocorticoid receptor (NR3C1) activates the stress response, reducing the activation of pro-inflammatory factors such as NF-κB and MAPK [47,48] (Figure 3A). Although usually well tolerated, considerations of side effects related to the hypothalamic-pituitary-adrenal axis after long term use, such as growth inhibition in children, increased risk of osteoporosis, adrenal inhibition, and diabetes, must be carefully balanced with any perceived benefit to exacerbation control [45,46,49]. Additionally, airway irritation may reduce compliance with medication schedules [50]. This class of drugs is usually paired with a long-acting agent for maintenance of low-to-moderate severity asthma, with the FACET (Formoterol and Corticosteroids Establishing Therapy) and GOAL (Gaining Optimal Asthma Control) studies confirming the synergy of paired therapy in 3742 total patients [37]. This effect occurs due to upregulation of the β2-adrenergic receptor by ICS, increasing the effectiveness of long-acting β2 agonists and (over the long term) minimizing ICS dosages to better prevent undesirable side effects [45].

### 3.2. Theophylline

Theophylline, known as dimethylxanthine, is a venerable medication in use for over 80 years that relieves airway inflammation through phosphodiesterase (PDE3) and PDE4-mediated increases of histone deacetylase 2 (HDAC2) that inactivate inflammatory genes, such as AP-1 or NF-κB, and downstream effectors, like interleukin (IL)-17A, through deacetylation of their subunits (Table 1) (Figure 3B) [51,52]. Additionally, as HDAC2 is involved in upregulation and stabilization of the glucocorticoid receptor (GR) responsible for the inhibition of inflammation (via MAPK and direct inhibition of interleukin transcription), HDAC2 increase through theophylline treatment may thus help to restore corticosteroid sensitivity [53,54,55]. However, due to side effects that include arrythmias (through deacetylation of the adeno-A1-receptor), seizures, nausea, and headaches, theophylline has been relegated to a support role for other, more specific controllers of bronchial inflammation, such as the long-acting beta agonists or ICS [39,51]. 

### 3.3. Long-Acting Beta Agonists/Muscarinic Antagonists

Beta adrenergic receptors found in the bronchial airway cause bronchoconstriction during an asthma attack, an effect countered by long-acting beta agonists (LABA) that promote bronchodilation in a synergistic action with ICS (Table 1) [56,57]. These combinations have been reported to decrease the Th2 inflammatory response by suppressing thymic stromal lymphopoietin (TSLP) mRNA in the bronchi (Figure 3C) [57]. Moreover, LABA upregulates endogenous steroid transcription, leading to an increased expression of the bronchoprotective gene RGS2, resulting in better asthma control [48]. Short-acting β2-agonists exist and are preferred for adolescents to regain control while avoiding the side effects of LABA (seen in multiple clinical trials) that include increased exacerbation risk and death [58]. Long-acting muscarinic antagonists (LAMA), or anticholinergics that can interact with and selectively block muscarinic acetylcholine receptors and dilate the bronchii, can also be used simultaneously with LABA [59]. 

### 3.4. Leukotriene Receptor Antagonists

Leukotrienes are lipid mediators derived from arachidonic acid that cause inflammation during allergic reactions, especially in neutrophilic or aspirin-induced asthma cases [60,61]. As an example, leukotriene B4 is a proinflammatory mediator that triggers neutrophil and eosinophil release into bronchial fluid while studies have found that it also crucially activates memory-T cells that mediate allergic airway reactivity (Table 1) (Figure 3D) [62]. These drugs are useful in children for whom ICS are not appropriate or for poorly controlled cases [58]. They are also useful for preventing exacerbations from exposure to cold or viruses with the caveat that liver condition must be carefully monitored [63].

### 3.5. Antibody-Derived Therapeutics

Monoclonal antibody-derived treatments (called biologics in view of their origin) specific for cytokine receptors that transduce allergic activation signals and reduce side effects through specificity are considered a new frontier of asthma therapy. Diverse anti-cytokine drugs, such as anti-TSLP antibody (tezepelumab), anti-IL-33R antibody (CNTO 7160), anti-IL-4Rα antibody (dupilumab), and anti-IL-5 antibody (mepolizumab and reslizumab), have gone or are currently undergoing clinical trials (Table 1) [46]. However, while IL-5-targeted mepolizumab and reslizumab were confirmed by the US Food and Drug Administration as treatments for severe asthma, other results are controversial and extensively reviewed elsewhere [16,64]. Multiple new biologics are also currently under development and are in clinical trials [65]. 

IgE is a key factor responsible for the degranulation of basophils and mast cells. Recently, human anti-IgE antibodies (omalizumab) were developed to prevent airway remodeling by suppressing inflammatory cytokines IL-6, IL-8, TNF-α, and IL-4 [66,67] (Figure 3E). TSLP, which increases sputum and eosinophils in asthmatics, is also an attractive target, since anti-TSLP antibodies may decrease bronchial constriction as well as airway thickness [68] (Figure 3E). Similarly, IL-33 receptor-targeted antibodies prevent Th2 cell-induced allergic reactions, and anti-IL-4Rα antibodies have improved FEV1 and asthma control, as well as decreased risk of asthma exacerbations, in several clinical trials [69,70]. 

### 3.6. Anti-Histamines and Anti-Allergics

Histamines are chemokines released from mast cells after IgE activation that cause smooth muscle contraction, mucosal edema, and bronchial discharge via Th2 response shifting [71,72]. Controlling degranulation via prevention of IgE production (such as with omalizumab) is useful but specific blockers of the histamine class of receptors, especially H1R or H2R, can globally downregulate lung inflammation and mucus secretion by interrupting histamine signal transduction [71,72] (Figure 3F). Additionally, since histamine mediates acid secretion in the stomach and there is a putative relationship between gastroesophageal reflux disease (GERD) and asthmatic exacerbations, proton pump inhibitors, such as cemitidine, may be useful to minimize reactive secretion of epithelial mucus, lung scarring, and facilitate bronchodilation, as well as reduce GERD-mediated airway irritation [72,73,74]. Loratidine or cetirizine, commonly prescribed for hay fever, have also shown benefits in clinical studies, as these medications are well tolerated as adjunct therapies. However, some antihistamines cause drowsiness, and others, such as terfenadine, have been associated with fatal cardiac arrythmias, and are banned in some countries [75,76,77,78,79].

### 3.7. Comparisons and Efficacy between Traditional and Biologic Treatments

Traditionally, corticosteroids (inhaled, oral, or injected as appropriate) are used to both treat emergency exacerbations, control inflammation, and reduce future exacerbations [80]. Additional treatments to address specific issues may see drugs, such as antihistamines, used in an adjunct fashion. However, the new class of biologics specific for interleukins and checkpoint proteins in the airway inflammatory pathway are a promising new possibility for both control and prevention of exacerbations without severe systemic side effects. These drugs, where approved, can be used alongside ICS or even oral corticosteroids for recalcitrant cases [81]. 

Within the biologics themselves, a networked meta-analysis of 30 Phase II or III trials conducted between 2005 and 2018 found that, while mepolizumab, reslizumab, and benralizumab were able to reduce exacerbation risk by around 20–50%, no single biologic was more effective than any other [65,82]. This collective effectiveness compares with ICS-LABA in a meta-review of budenoside/formoterol where overall severe exacerbation reductions of around 26% were observed, in line with a 1997 study and the GOAL and FACET studies [37,83,84]. However, with the exception of benralizumab, some studies do not reach sufficient statistical power to make concrete determinations of biologic utility for all cases [82]. Moreover, there are patient phenotypes featuring a distinct lack of response biomarkers that are unresponsive even to biologics and these patients may also be resistant to typical ICS+LABA therapy [85]. But, if the patient has a responsive phenotype, combinations of ICS with biologics or even biologics alone could provide reductions in exacerbation with relatively fewer side effects. The growth stunting and other systemic side effects seen in children on long-term ICS make biologics attractive in this regard, due to very few serious drug reactions, but reduced response to vaccinations and increased infection risk must be considered [49,86]. 

## 4. Lung-Focused, Mechanical Drug Delivery Systems 

Since asthma is a respiratory disease, logic posits that providing therapies directly into the lungs/pulmonary system would more effectively block flare ups, as well as provide rapid and sustained relief from exacerbations. Inhaled delivery also allows for bypassing of the first-pass liver metabolism. However, the lungs are a complex system, engineered to provide a barrier between particulates suspended in the air and bloodstream. Additionally, co-morbidities, such as chronic obstructive pulmonary disease (COPD) may limit the absorption of inhaled medications. Moreover, studies have shown that rescue and asthma control inhaler types should not be mixed to achieve optimal results and that any switch in inhaler type should be done carefully to ensure medication compliance [87,88]. Thus, multiple inhaled medications are often necessary to achieve control of and relief from exacerbations. Of prime importance to inhaled medications is the dosage routine, which requires training and willful compliance on the part of patients as low education levels, socioeconomic concerns, and age may result in delivery errors or noncompliance that result in poor control of asthma or COPD complicated by asthma [89,90,91]. A summary of delivery systems designed to overcome these challenges and the benefits/drawbacks of each is summarized in Table 2.

### 4.1. Propellant-Pressurized Metered Dose Inhalers

The propellant-pressurized metered dose inhaler (pMDI) consists of a small tank of pressurized, liquid propellant (such as chlorofluorocarbons in the earliest versions and now hydrofluoroalkanes or other inert gasses) and a drug that readily suspends in the liquid propellant [92]. Upon release, the high vapor propellant rapidly forms a gas that carries fine particles (5 microns or less) of the drug suspension into the airway, with finer particles (on the order of 500 nanometers) providing the best deposition into the deep lungs [93]. Since their first use in the 1950s, multiple types, such as the press-and-inhale, velocity-modified, breath-coordinated, and breath-actuated, are available to fit a wide range of patient needs [94].

The pMDI delivery system carries the advantages of precise delivery, as a mechanical valve and the reliable propellant action cooperate to standardize each metered dose. Studies have shown similar efficacy as dry powder inhalers, while the self-contained propellant is optimal for COPD patients, or those who have very limited inspiratory ability [95,96]. Newer, breath-triggered types that dispense with the normal breathing rhythm remove the need for deep inhales, and may be suitable for children [96]. Of importance in wet climates, the completely enclosed and airtight system keeps out water and humidity. On the other hand, pMDI systems are not always used properly, and some patients may need spacers or training to coordinate inhalation with dispensing [97,98]. Additionally, propellants (such as norflurane) may have anesthetic properties, relaxing the smooth muscles and adding unaccounted for synergy to the effect of the medication [99]. Standard hazards of pressurized containers (such as rupture) are also a concern while rare studies on the effect of altitude on dosing has found that some inhalation units may underdeliver at high altitudes, which is undesirable since the colder air found at such altitudes tends to exacerbate asthma [100,101].

### 4.2. Dry Powder Inhalers

The dry powder inhaler (DPI) is a particle delivery system that mixes drugs with a powdered carrier (such as lactose) and dispenses a precise amount to be uptaken upon deep inspiration [102]. Simple and cheap to manufacture, the DPI requires no pressurized gasses, complex switching mechanisms, or electricity to operate. Various methods to dispense the drug have been developed, including spring-actuated mechanisms, sliding plastic parts, and reservoirs for multiple doses in one container [102]. 

Thanks to their low manufacturing complexity and the fact that some are refillable, these devices may be an attractive option for healthcare systems that seek to minimize cost [103]. Since no propellant is needed, altitude concerns are negated and poorly soluble drugs can be more easily bound to a solid carrier [102]. DPIs also offer the benefit of rapid action as diffusion into the bloodstream is contact-based and no other vapors or gasses are present to impede drug-surfactant contact or trigger secondary effects [104]. However, studies have shown that compliance with DPI inhalers is lower than pMDI, possibly due to annoyance at the required breath holds (3 s or more), and switching to pMDI from DPI reduced exacerbations in a Korean study population [105]. Furthermore, a study of 111 patients showed better control with pMDI, since the larger particle size of DPI dispensers may not allow for sufficient penetration into the deep lungs and reports have indicated that DPI dispensing precision relies on optimal humidity and electrostatic conditions [36,106,107]. 

### 4.3. Nebulizers

Nebulizers are a delivery system based around either the oscillating movement of a membrane by a piezo-electric actuator, an ultra-fine mesh to aerosolize liquids, or a Bernoulli-style gas pressure (air or oxygen) differential flow system [108]. These systems must be powered, either by electricity or pressurized gas, and come in a variety of large and small tabletop units, as well as battery-powered portable units. 

Nebulizers are an effective form of treatment that rely on normal breathing patterns with no special patient training needed, and are often used for recalcitrant or difficult-to-control asthma since continuous delivery of diverse medication types can be maintained over long periods of times. Reports have demonstrated the efficacy of home nebulizer treatment for poorly controlled asthma, especially in young children for whom inhalers may be misused due to an inability to understand proper inhalation methods, while new formulations of liposomal or micro-particulate suspensions may increase delivery efficiency [109,110]. Additionally, the ability to finely tune particle size and the precision of delivery make it attractive for clinical usage, especially in the infirm or those with an inability to deeply inhale or comply with the coordinated breathing needed for pMDI or DPI systems [111]. Nebulizers are not, however, the perfect system, as they are bulky (due to requirements for batteries or continuous gas pressure), require extended periods of time to dispense a bolus of medication (versus instant delivery through a pMDI or DPI system), and may shear or damage microencapsulated medicines before delivery [112]. New types of nebulizers are in testing that attempt to overcome traditional limitations by delivering drugs without this shearing effect [111,112].

### 4.4. Comparisons between Delivery Systems

The pMDI system is preferred to DPI systems, as it delivers into the deep lungs without upper airway deposition and avoids bacterial contamination due its sealed, pressurized, and unidirectional flow design [113]. However, a large European study on a new DPI triple therapy featuring extra fine granules found no difference between DPI and pMDI in 366 randomized patients [114]. This result was not seen in a UK study of 1567 pMDI vs. 1567 DPI patients in which pMDI patients experienced better asthma control and treatment success, even if the exacerbation rates did not significantly differ [115]. 

Nebulizers are usually reserved for emergency treatments during acute attacks, but a study in 1998 found that, when adjusted to deliver the same amount of salbuterol to the deep lungs, no significant differences were found between nebulizer and pMDI systems [116,117]. A Cochrane review of 39 randomized trials (with a total of 1897 juvenile and 729 adult patients) in 2013 found that nebulizers were not superior to pMDI and that, if appropriate spacers were used, the pMDI users had emergency room stays of up to 33 min shorter [118]. 

In spite of any perceived advantages, DPI and nebulizer systems have been shown in literature reports to be less effective (or no more effective) than pMDI systems and, thus, pMDI delivery with appropriate spacers seems recommended in the literature for delivery of both control and relief medications. 

## 5. Drug Packaging

The challenging aspect of asthma drug synthesis is the requirement for rapid and long action. Usually, any oral drug first passes through the liver and the cytochrome system; this first-pass metabolism reduces the amount of compound available and modifications to reactive side groups may change the activity or targeting of the active ingredient. However, current formulations may include extended release modifications (enteric coatings, osmotic reservoir, hydrophobic/philic co-matrices, etc.) to keep serum levels high over many minutes or hours [119,120]. This metabolic assimilation takes time, however, and rapid relief of asthmatic symptoms usually requires direct delivery to the airway. Inhalation, on the other hand, delivers directly to the target epithelium in the bronchi and alveoli while maintaining an acceptable half life of 2 to about 14 h [121]. However, sometimes these drugs require larger doses (up to quadruple in a recent study) to control exacerbations, increasing the risk of local and systemic (via hypothalamic–pituitary–adrenal axis) side effects [122,123]. 

### 5.1. Use of Nanoparticles for Delivery of Therapeutic Agents in Asthma

**Figure 4:**
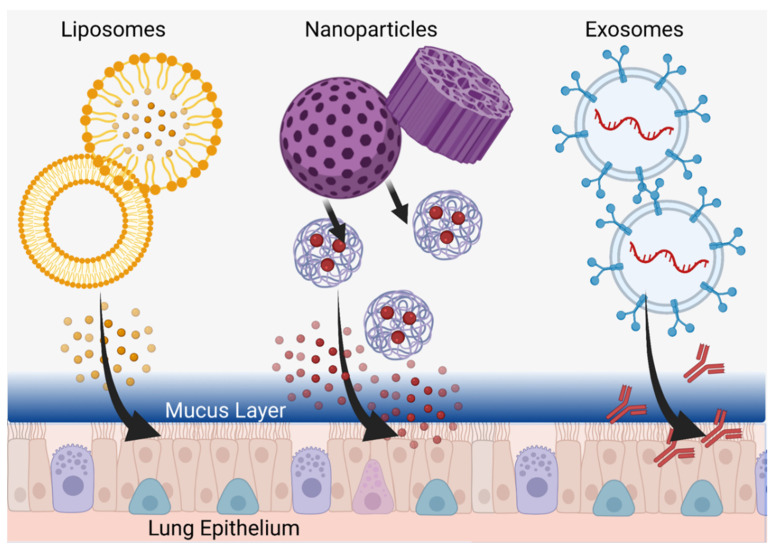
Customized Delivery Systems. Liposomes (**left**), nanoparticles (**center**), and exosomes (**right**) can be tailored to penetrate into the deepest parts of the lung and deliver therapeutics directly to epithelial cells through the mucus layer. Created in BioRender.com.

Due to their potential for more effective delivery of active therapeutic agents to target cells and organs, diverse nanoparticle types, including nanoscale liposomes, are a promising translational research subject. With their larger size and greater capacity than low-molecular-weight compounds, nanoparticles are capable of delivering not only much larger amounts of active components, but also doing so in a more technologically intelligent system that can be engineered based on desired release criteria. A new frontier is thus the development of micro/nanoscale delivery complexes which, in addition to active ingredient transport, can overcome physiological barriers, as well as actively target specific cells in the human body. This is a challenging but very necessary priority in cases of treatment resistance, where disease control remains unsatisfactory in some patients who do not fit the immunological profile to use more specific additional therapeutic methods (such as monoclonal antibodies). Several types of micro/nanoscale agents have been identified and tested for the treatment of asthma, including synthesized liposomes and nanoparticles, as well as naturally produced nanoscale extracellular vesicles (Figure 4).

### 5.2. Liposomes

Liposomes are spherical nanoscale structures based on a bilipid layer that delimits the internal space from the external environment, helping to securely encapsulate a relatively large internal volume of active components and limit their undesirable effects on organs and tissues not involved in therapeutic targeting (Figure 4) [124]. Liposomes have been used in clinical practice since the 1990s when Doxil^®^, a liposome-encapsulated doxorubicin drug, was first approved [125]. Liposomes have also become clinically applicable for the delivery of anti-inflammatories, analgesics, antifungals, antitumor drugs, in gene therapy, and for the creation of antiviral vaccines [124]. Thus, among the various types of nanoparticles, liposomes are the first to claim clinical usage in asthma patients. However, the first liposome-based drugs were modeled for intravenous or intramuscular administration (in the case of vaccines). As an example, the characteristic enhanced permeability and retention (EPR) effect and the associated delivery of liposomes through damaged biological barriers in tumor tissue may be most actively used in oncology to overcome tumor barriers but this requires blood delivery [126]. In the case of asthma, on the other hand, the inhalation of liposomes is desired, encountering fundamentally different physiological barriers versus traditional intravenous, intramuscular, or intra-arterial administration methods. In this case, reaching the surface cells of the pulmonary epithelium, as well as the basal cells, will be complicated for any liposomes by both a mucosal barrier of glycosylated mucins and an immune barrier of alveolar macrophages [127]. Such biological barriers should be taken into account in the development of anti-asthmatic liposomal drugs and, currently, in vitro and in vivo studies are being conducted to test liposome-encapsulated drugs for their efficacy in animal disease models. 

The chief goals of liposome use are to maintain a longer therapeutic concentration of agents (including currently prescribed drugs), the time of their persistence in the lungs, and the therapeutic effect associated with them. For example, in recent studies by Chen at al. in which a new, aerosolized liposomal form of salbutamol sulfate for intratracheal administration underwent an in vitro permeation study in the pulmonary membranes of Asian toads, slower liposome transport was observed compared with the non-liposomal form of the drug [128]. When administered intratracheally in rats, drug release was maintained for 48 h or more and the anti-asthmatic effect of the liposomal form in a guinea pig model was noted up to 18 h, compared with the 8-h effect of the conventional drug form [128].

In 1997, a clinical study was conducted on a group of 78 patients (aged 18 to 63 years) with steroid-dependent severe bronchial asthma in which a liposomal form of hydrocortisone was used for intratracheal irrigation of the bronchi during bronchoscopy [129]. The authors performed a complete clinical and immunological examination of the patients and found that intratracheal use of liposomes with hydrocortisone contributed to the restoration of normal indicators of humoral and cellular immunity as well as the relief of inflammation in the trachea and bronchi [129]. However, the most pronounced effect was in the combined therapy group, where patients received drugs in both liposomal form intratracheally and standard form systemically [129].

Given that biological membranes play an essential role in the assimilation of inhaled anti-asthmatic drugs, studying such biochemical interactions can pave the way to new therapeutics. The ability of cell membranes to change the pharmacological activity of drugs, the behavior of the drug in liposomes, and relationships with liposomal components are all priority areas of study in this regard. For example, the activity of the β2-adrenoreceptor agonist drug terbutaline sulfate was studied using dimyristoyl-sn-glycero-3-phosphocholine (DMPC) or DMPC and cholesterol-containing liposomes, in which the membrane affinity of terbutaline sulfate was confirmed by the location of the drug in the membrane, its effect on membrane fluidity, and changes in the partition coefficient [130]. The composition of the liposomes, in turn, influenced the degree of this affinity which favored DMPC-only membranes more than combinations of DMPC and cholesterol [130]. 

Interaction studies of liposomes and immune cells also contribute to a better understanding of the local metabolism occurring in the lungs after the introduction of new drugs. An early study, done by Myers et al., showed the possibility of using liposomes in aerosol form for inhalation administration [131]. They investigated the uptake of liposomes, consisting of hydrogenated soybean phosphatidylcholine (HSPC), by alveolar macrophages in mice, showing that a prolonged presence of liposomes in the lungs resulted in no deleterious changes in general condition, tissue, or phagocytic functionality [131]. 

Given that viral infections can cause exacerbations in chronic inflammatory lung diseases, including asthma, stimulation of the antiviral cytokine IFN-β can be used to control such exacerbations. Dauletbaev et al. used liposomal polyinosin-polycytidylic acid [poly(I:C)] to induce IFN-β and observed reductions in associated inflammatory responses [132]. In vitro, the authors showed that the use of the liposomal form of poly(I:C) promoted IFN-β stimulation through the RIG-I/MAVS pathway under low stimulation of IL-8 in contrast to the non-liposomal form of the drug, where IFN-β was stimulated through the TLR3/TRIF pathway under IL-8 co-stimulation [132]. The results of this study, along with others, emphasize the advantages of liposomal delivery of active ingredients and further justifies the development of liposomal drug forms for asthma treatment.

### 5.3. Nanoparticles

Studying the effect of nanoparticles with incorporated active ingredients for the treatment of asthma, just as with liposomes, allows new vectors for delivery technology. In contrast to other areas of biology and medicine, such as oncology and radiology, where metallic nanoparticles (particularly gold) enhance therapeutic effect and diagnosis, the systemic absorption of these in long-term asthma use leads to side effects and thus emphasizes the use of biodegradable, non-metallic nanoparticles [133,134,135]. Lee at al. synthesized such non-metallic dexamethasone nanoparticles with peroxide-activated boronate-maltodextrin, releasing dexamethasone and reducing inflammation by suppression of inflammation-inducing cytokines in both macrophages and lung epithelium of BALB/c mice (with allergic asthma) at doses of 5 mg/kg without obvious side effects [136]. Using a rat model, Mohamed et al. developed lipid-polymer hybrid nanoparticles in a thermosensitive gel for intranasal delivery of terbutaline sulfate to improve its bioavailability and increase nasal residence time, demonstrating three times greater permeability of the drug in the nanoparticles through the nasal mucosa (compared to the free form) as well as achieving favorable outcomes in tidal volume and peak expiratory flow [137]. These reports bolster the utility of non-metallic nanoparticles for mucosal delivery, a system quite amenable to the inhalation requirements of long-term asthma medications (Figure 4). 

The most promising developments in this area are the creation of nanoparticles based on polymers, which are already approved for clinical use. For example, Chakraborty et al. proposed the use of andrographolide (AG), which has anti-asthmatic potential and relatively low toxicity, in biodegradable polylactide-co-glycolide (PLGA)-based nanoparticles [138]. Full pharmacokinetics and biodistribution tests of the nanoparticles, with oral and inhaled routes of administration, were studied in murine models, showing sustained drug release of the drug in vitro (effective encapsulation of 5.47%), higher in vivo bioavailability, more significant reduction in inflammatory cells (decreased IL-4, IL-5 and IL-13 in lung lavage fluid), lower serum IgE levels, and more pronounced NF-κB suppression using nanoparticles in the pulmonary route of administration [138].

The development of new active ingredients for incorporation into these existing nanoparticles are also of interest. In this regard, Morris et al. proposed the inhibition of inflammation-mediating Ca^2+^/calmodulin-dependent protein kinase (CaMKII), localized in the bronchial epithelium of asthma patients, by inhaled administration of polylactide-co-glycolide (PLGA)-based cationic nanoparticles with CaMKII-inhibitory peptide (CaMKIIN) and demonstrated a reduction in the severity of allergic asthma in a mouse model [139]. Of note, the proposed modification of the nanoparticle surface with chitosan was even more effective in increasing drug uptake by airway epithelial cells [139]. Lv et al. synthesized chitosan-based nanoparticles for intranasal inhalation of recombinant interleukin-17 C receptor protein (rIL-17RC) and showed significant inhibition of mucus secretion and airway infiltration by inflammatory cells as well as decreases in IL-4, IL-17, IL-17F levels in bronchoalveolar lavage fluid [140]. Thus, chitosan looks promising as a nanoparticle material for clinical development.

Despite the previously described limitations of metal nanoparticles, a report on mediation of group 2 innate lymphoid cells (ILC2s) involved in asthma pathogenesis (via inhibition of suppression of tumorigenicity 2 [ST2]) demonstrated the effectiveness of superparamagnetic nanoparticles of iron oxides (SPIO) [141]. These were conjugated with anti-ST2, reducing lung inflammation in a BALB/c mouse model by reducing IL-5 and IL-13 production by ILC2s and subsequently reducing the number of CD4+ T cells [141]. This report emphasized that the increase in anti-ST2 efficacy was achieved with nanoparticles without specifically mentioning SPIO nanoparticles; however, such results may lend weight to the use of metallic nanoparticles for short-term control of inflammation associated with asthmatic exacerbations.

Additional reports with diverse combinations of nanoparticles and pharmaceutics have demonstrated some effectiveness. A notable report on the efficacy of inhaled gene therapy to completely eliminate the key pathology of allergic asthma in mice used nanoparticles with thymulin-expressing plasmids [142]. The results detailed the ability of nanoparticles to penetrate the mucosal airway barrier and reverse the biochemical, histological and functional manifestations of allergic asthma by modulating eosinophils, neutrophils, and lymphocytes/macrophages, thus normalizing chronic inflammation, pulmonary fibrosis, and mechanical dysregulation [142]. Additionally, Luo et al. also used PLGA to synthesize nanoparticles containing A20, or tumor necrosis factor alpha-induced protein 3 (TNFAIP3), and ovalbumin (OVA) to form an intranasal PLGA-OVA+A20 nanovaccine, which relieved allergic asthma in BALB/c mice by increasing production of Treg cells better than the free form of OVA+A20 [143]. Taken together, these reports outline the versatility and vast customizability of nanoparticles in the treatment of asthma, especially for those patients somehow unable to receive standard monoclonal therapies.

### 5.4. Extracellular Vesicles

Another direction in the development of asthma therapy with nanoscale materials is the use of natural membrane particles, or extracellular vesicles secreted by various cells, that mediate intercellular communication important to the immune system response [144,145]. These particles, including microvesicles and nanoscale exosomes, are capable of encapsulating the proteins, lipid mediators, and nucleic acids (including miRNA) that play key roles in the development of pathological processes such as allergic asthma [145,146]. Moreover, these vesicles can serve both as biomarkers to help distinguish different types of allergies in patients and as particles to deliver active molecules that suppress further asthmatic pathogenesis and exacerbations [147,148,149]. 

Extracellular vesicles themselves can be characterized by low immunogenicity and high safety in biological systems while, in terms of modulating agent delivery, nanosized exosomes may be more attractive due to their small size and potential to penetrate the pores of the mucus layer and transmit signals directly to airway epithelial cells (Figure 4) [150]. On the other hand, due to air flow principles, the use of bulkier and heavier microparticles during inhalation administration may facilitate more effective delivery to remote parts of the lungs. In this regard, combined structures could be used where microparticles will act as delivery modules for smaller nanoparticles, which, upon deployment, will be able to penetrate deep into the epithelium through the thick mucus layer [127,151].

The efficacy of extracellular vesicles synthesized by mesenchymal stem cells (MSCs) has been demonstrated in diverse reports. Ren at al. showed the immunomodulatory effect of exosomes derived from mesenchymal stem cells (MSC-Exo) in a BALB/c induced asthma model where, after intranasal injection of exosomes, the number of interstitial macrophages and their IL-10 levels significantly increased in the lungs, contributing to attenuation of allergic airway inflammation [149]. In experiments using splenectomized mice and conditionally deficient mice with IL-10-specific Cx3cr1 + cells, the authors concluded that protection depended on IL-10 produced by interstitial macrophages probably originating from the spleen [149]. As for smaller-sized particles, Fang et al. developed a protocol for isolation of MSC-small extracellular vesicles (sEV) by anion-exchange chromatography and showed that MSC-sEV prevented allergic airway inflammation in ILC2-dominant mice with partial involvement of miR-146a-5p [150]. In translational studies, Dong et al. found that human umbilical cord MSCs (hUCMSCs) can secrete more extracellular vesicles under hypoxia than normoxia and these had a more pronounced airway inflammation suppression effect in a mouse model of chronic asthma with additional prevention of chronic allergic airway remodeling by reductions in profibrogenic α-smooth muscle actin (α-SMA), collagen-1, and TGF-β1-p-smad2/3 signaling pathways [148]. Draijer et al. showed that extracellular vesicles synthesized by resident alveolar macrophages, containing cytokine signaling suppressor 3 (SOCS3), could inhibit inflammatory signaling in alveolar epithelial cells while synthetic liposomes containing SOCS3 restore this inflammatory suppression effect when impaired in case of asthma [152]. Shang et al. demonstrated downregulation of mmu_circ_0001359 in asthmatic mice and showed that exosomes from adipose-derived stem cells modified by mmu_circ_0001359 reduce airway remodeling by activating M2-like macrophages via enhanced FoxO1 signaling and miR-183-5p sponging [153]. In another lung-focused study, Wu et al. showed that extracellular vesicles synthesized by hemin-primed dendritic cells could reduce mucus secretion, eosinophil infiltration, IL-4, IL-5, and IL-13 levels in the lungs (along with Th2 cell numbers) while increasing Treg cells in mediastinal lymph nodes, thus alleviating allergic airway inflammation in a murine asthma model [154].

MicroRNA delivery by exosomes, in particular, has been studied for specificity of action against asthmatic inflammation. Zhuansun et al. showed that miR-1470 contained in mesenchymal stem cell exosomes can induce the expression of P27KIP1 and promote the differentiation of CD4 + CD25 + FOXP3 + Tregs in patients with asthma [155]. Li et al. determined that miR-21-5p-containing exosomes produced by rat macrophages are able to transfer miR-21-5p to rat airway cells by promoting epithelial-mesenchymal transition of these cells via the TGF-β1/Smad signaling pathway by targeting Smad7 [156]. In a recent review, Carnino et al. summarized the results of diverse experiments showing the role of extracellular vesicles with microRNAs capable of transporting genetic material and transferring it to epithelial cells in altered lungs under various chronic disease conditions, including asthma [157].

The effect of exosomes secreted by eosinophils, which can influence remodeling in asthma by activating lung structural cells, has also been studied [158]. Additionally, exosomes from human macrophages and dendritic cells containing functional molecules that promote leukotriene synthesis were shown to promote granulocyte migration and inflammation [159]. Understanding such mechanisms may serve as a prerequisite for the development of new methods of asthma suppression that involve exosomes. 

As the number of exosomes used in reported experiments may be relatively small, the use of such a physiologically relevant amount may be sufficient to show an effect. However, for larger clinical applications, more extensive technologies for synthesizing these agents are required and multiple techniques have been proposed and tested for producing synthetic exosomes in quantities sufficient for larger-scale applications [150,160,161,162]. Jo et al. proposed a new method for creating artificial vesicles resembling cell-secreted exosomes by passing cells through hydrophilic microchannels (in which exosomes can contain mRNA, intracellular proteins, and plasma membrane proteins) that are subsequently transferred to target cells [161]. The same group of authors also proposed the use of a centrifugal force device with a filter featuring microdimensional pores which makes it possible to obtain approximately 250 times more nanovesicles, each containing twice as much RNA and protein compared to natural synthesis [160]. 

Thus, natural and synthetic exosomes are on par with liposomes and nanoparticles and may even exceed the capabilities of the latter in the application of nanomaterials for new treatment delivery systems in allergic asthma and other pulmonary diseases.

### 5.5. New Horizons of Delivery Potential

**Figure 5:**
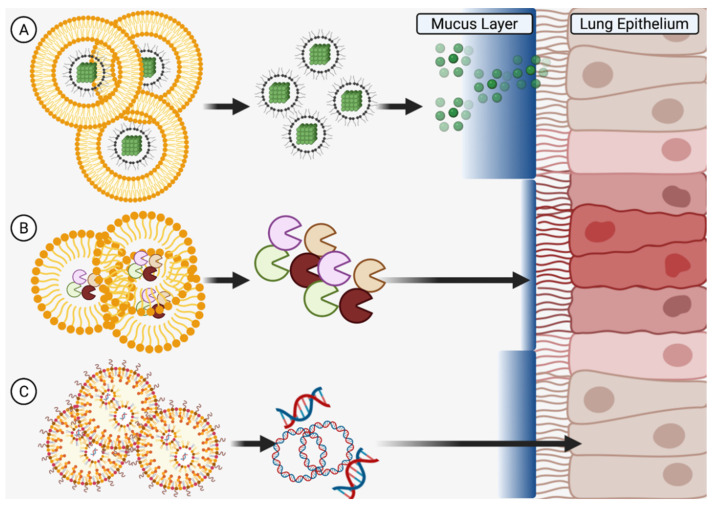
New Horizons in Delivery Potential. Microscale structures can be engineered to (**A**) carry nanoscale particles deep into the lungs and past the mucous barrier, (**B**) release enzymes to locally modify the mucous for easier penetration of subsequent medicines, or (**C**) release gene therapy materials to change mucous expression profiles. Created in BioRender.com.

The development of more advanced nanocarriers of anti-asthmatic compounds carries several possibilities related to inhaled delivery and, in particular, full penetration into the deepest lung compartments. In this regard, since air flow can carry heavier microparticles deeper in the lungs compared to lighter nanoparticles or liposomes (that sediment closer to the trachea and bronchi), more complex microparticles serving as carriers for nanoparticles that are released closer to the epithelial cells might partly solve this issue (Figure 5A) [163]. Another option relates to the efficacy of penetrating the thick bronchial mucous barrier, which might be solved by the inclusion of specific enzyme-like (or other biocompatible) molecules on the nanoparticle surface that can locally modify the mucus to provide a direct path to the epithelial cells (Figure 5B) [164]. Alternative guidance of nanoparticles to epithelial cells might also be achieved through small molecules targeting proteins on the cell surface or through active particle migration with further recognition of the targeted cells. 

Apart from carrier-specific delivery options, the active compounds carried by nanoparticles could use specific substances, such as nanoliposomes or polyethyleneimine, to carry material that influences epithelial gene expression (by gene therapy using nucleic acids) to modify mucus characteristics and/or synthesis (Figure 5C) [142,163]. Advancements such as these might provide an alternative, more efficient way of coping with allergic asthma in patients with genetic profiles unsuitable for monoclonal antibody therapy. However, it is critical to also consider the aftermath of delivery as particulate accumulation in the lungs (even on a nanoscale level) in long-term users may result in side effects such as inflammation or fibrosis [164].

## 6. Requirements for Effectiveness in a Target Population

### 6.1. Nanoscale Delivery Parameters for Drug Delivery in Children

Asthma is common in adolescents of all ages and ethnicities, and may stem from both genetic and environmental factors that result in hypersensitivity of the developing immune system [165,166]. As childhood asthma has been reported to stem from diverse sources, including atopic family history, pollution exposure, microbial burden, polymorphisms affecting T cells, and passive smoking, efforts to prevent have given way to therapies to control exacerbations and provide long-lasting relief [166]. Although first-line ICS+LABA therapies are fairly effective in children, corticosteroids have been reported to stunt growth while effective biologic drugs based on IgE-neutralizing monoclonal antibodies show promise for longer control but also carry risks of adverse events [123,167,168,169]. Thus, delivery systems built around hydrogels could isolate and keep ICS from the general circulation and liposomal systems for biologics may allow for rapid absorption into airway epithelium before diffusion into the bloodstream can occur (Table 3). Polymeric or diffusion-based systems (liposomal or nanoparticle) may also be useful to keep serum levels of anti-inflammatory therapeutics constant without frequent dosing, a consideration when dealing with the high rates of medication noncompliance seen in adolescents [170]. 

### 6.2. Nanoscale Delivery Parameters for Drug Delivery in Athletes 

An athlete’s measures of fitness, performance, and success revolve entirely around the ability to keep blood oxygen levels high and utilize that oxygen efficiently. Thus, athletes with asthma face both physiological barriers to competition (exacerbations) as well as legal restraints (anti-doping rules) on the types and varieties of therapies they are allowed to take during competitions [171]. For example, some LABA are prohibited during competitions since their relaxing effect on smooth muscle could increase oxygen uptake and impart a decisive blood oxygen advantage over competitors [172]. Therapies for competing athletes, therefore, should have a short but rapid action and be easy to detect in screening. Liposomal formulations may be ideal for this as they would keep systemic effects to a minimum by increasing absorption speed in the epithelium while inhaled nanoparticulate carriers may offer testing benefits as the carrier itself could be detected in drug screening (Table 3). Transdermal patches or microneedles delivering exosomal therapies might also be useful in that the diffusion and uptake rates of such patches can be well-controlled (especially by electrical devices that dispense via microneedles) and extraction of analytical fluids through the patch for screening purposes is also possible [173]. 

### 6.3. Nanoscale Delivery Parameters for Drug Delivery in Co-Morbid Conditions

**Figure 6:**
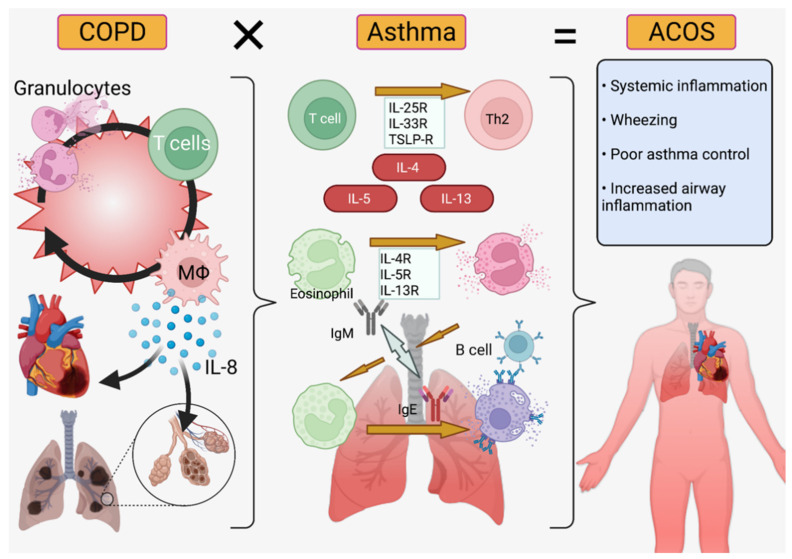
Asthma-COPD Overlap Syndrome (ACOS). Chronic obstructive pulmonary disorder (**left**) is primarily IL-8/neutrophil mediated while asthma (**center**) is IL-4/5/13 and T-cell mediated ACOS (**right**) features symptoms from both diseases and requires increased inflammation control [16,17,176]. Created in BioRender.com.

COPD is a terminal disease featuring inflammation of the airways and, because of overlapping molecular mechanisms, asthma therapies are often useful to keep airways open. However, COPD patients have reduced inspirative capacity, reduced immune regulatory ability, and a biochemical pathogenesis centered around IL-8, CD8+ T cells, neutrophils, and macrophages versus the IL4-, IL-5, IL-13, and CD4+ T cell-driven phenotype of asthmatics (Figure 6) [16]. These patients thus require combinations of medicines, such as ICS-LABA-LAMA, that may cross-react at a chemical level or counteract each other at a systemic level [174]. Therefore, asthma-COPD patients, often said to have ACOS (asthma-COPD overlap syndrome, Figure 6), may benefit from nanocarriers that separate medicinal components while in solution as well as micellar or liposomal packaging that encapsulates drugs to prevent contact while in liquid propellant. These liposomal or micellar delivery systems may also increase solubility of drugs in propellants or solvents [175]. Biopolymer diffusion (e.g., chitin nanoparticles) may be useful for systemic administration of steroids to control inflammation while hydrogel delivery may assist in localization and retention of drugs in the lung epithelium. As most COPD patients are aged, they may have difficulty metabolizing the active components of traditional therapies while remaining highly susceptible to their systemic side effects, such as glaucoma or osteoporosis [121]. For this reason, inhaled nanoparticles targeting epithelial cells for delivery may reduce the susceptibility of these patients to systemic side effects while hydrogels would offer the additional advantage of long retention, preventing the need for frequent dosing and exposure to medications (Table 3). 

## 7. Conclusions

Asthma is a heterogenous, chronic affliction with a clear immune component but enigmatic pathogenesis. Since modern medicine has failed at prevention, it currently focuses on relief from and control of exacerbations. Diverse drugs exist for this purpose, from older bronchodilators to modern biologics derived from monoclonal antibodies. However, antiquated delivery systems hinder treatment through difficulties in use, noncompliance, and limitations in chemical or delivery parameters (e.g., batteries for nebulizers or solubility for inhaled compounds) that current packaging systems cannot overcome. New classes of chemical packaging/delivery, with transdermal, lipid-containing, nanoscale, receptor-specific, or other customizable and specialized properties, are being developed that may give new life to the use of older drugs in the fight against asthma. Clinical trials to evaluate the performance of these new delivery technologies will certainly increase the diversity and utility of the treatments in the physician’s toolbox. 

## Figures and Tables

**Table 1:** Representative Conventional Drugs for Asthma.

Drug Name (Generic)	Drug Type	Benefits	Disadvantages	Representative Clinical Trials
Beclomethasone dipropionate [36]	Inhaled corticosteroid	Reduces airway swelling	Dry mouth, irritated throat, voice changes	NCT02040766 NCT03834012 NCT00497237 NCT02513160
Fluticasone propionate [35]	Inhaled corticosteroid	Prevents allergic reactions	Nasal dryness, nausea, vomiting	NCT02175771 NCT02139644 NCT02301975 NCT02980133
Budesonide [37]	Inhaled corticosteroid	Reduces airway swelling	Dysphonia, oropharyngeal candidiasis	NCT01676987 NCT05152355 NCT01070888 NCT00509028
Ciclesonide [35]	Inhaled corticosteroid	Reduces airway swelling	Dysphonia, oropharyngeal candidiasis	NCT03839433 NCT00163293 NCT01455194 NCT00305461
Fluticasone furoate [35]	Inhaled corticosteroid	Reduces airway swelling	Dysphonia, oropharyngeal candidiasis	NCT01159912 NCT02502734 NCT01165138 NCT00649025
Mometasone [35]	Inhaled corticosteroid	Reduces airway swelling	Dysphonia, oropharyngeal candidiasis	NCT00556673 NCT02415179 NCT01555151 NCT01210170
Tiotropium [38]	Long acting muscarinic antagonist	Bronchodilator	Dry mouth, constipation	NCT03964220 NCT00557700 NCT02676089 NCT01340209 NCT00776984
Theophylline [39]	Xanthine Derivative	Relaxes airway muscles	Nausea, abdominal pain, headache, diarrhea	NCT01684683 NCT00119496 NCT00756418 NCT00000578
Salmeterol xinafoate [36]	Long acting beta agonist	Prevents bronchoconstriction	Hives, headache, blurred vision	NCT02260492 NCT03461627 NCT04564456 NCT03535870
Pranlukast hydrate [40]	Leukotriene receptor antagonist	Prevents bronchospasm	Headache, abdominal or stomach pain, cough, dental pain	NCT03826485
Montelukast sodium [40]	Leukotriene receptor antagonist	Prevents bronchospasm	Numbness, pain in the arms or legs, sinus pain	NCT00140881 NCT00157937 NCT00092989 NCT00636207
Mepolizumab [41]	Antibody derivative	Prevents airway swelling	Pain at injection site, headache, rash	NCT02281318 NCT01691521 NCT02555371 NCT02654145
Reslizumab [42]	Antibody derivative	Anti-eosinophilic activity	Pain at injection site, headache	NCT03074942 NCT02452190 NCT00587288 NCT03052725
Benralizumab [43]	Antibody derivative	Anti-eosinophilic activity	Pain at injection site, headache	NCT02322775 NCT02869438 NCT02075255 NCT02814643
Omalizumab [44]	Anti-IgE antibody	Prevents allergic reaction	Itching, bruising, redness, pain, or swelling at the injection site	NCT00314574 NCT01922037 NCT02654145 NCT00046748

**Table 2:** Summary of Asthma Treatment Delivery Systems.

Class of Delivery System	Delivery Method	Subtypes	Pros	Cons	Clinical Studies (Representative)
Propellant-Pressurized Metered Dose Inhalers (pMDI)	-Aerosolization via liquid propellant under pressure -Inhale deeply upon release	-Press-and-inhale -Velocity- modified -Breath-coordinated -Breath-actuated	-Precise and instant delivery -Waterproof -Pressure delivery	-Requires training -May be altitude sensitive -Tank can rupture -Spacer may be needed	NCT02091986 NCT01136382 NCT00746330 NCT01070524 NCT01803087
Dry Powder Inhalers (DPI)	-Metered dispensing -Deep inhale -Breath hold	-Spring-loaded -Multi-dose	-Simple -Refillable -Rapid action -Easy to formulate	-Particle size is not ultrafine -Vulnerable to humidity and electrostatic influence	NCT03478657 NCT02794480 NCT02753712 NCT01191424 NCT02022761
Nebulizers	-Aerosolization of liquid -Normal breathing	-Piezo-electric membrane -Bernoulli gas pressure differential -Ultrafine mesh	-Uses normal breathing pattern -Can mix drugs easily -Can fine tune particle size	-Bulky -Requires continuous pressure or electricity -Extended treatment time	NCT01951378 NCT01045174 NCT01649401 NCT03029156 NCT02774941

**Table 3:** Summary of Parameters Needed for Drug Delivery by Population.

Population	Condition	Requirements	Suitable Delivery Methods
Children	Developing immune system Growth stage	Moderate speed of action Long lasting Reduction in systemic steroid exposure	Inhaled (liposome) Diffusion Hydrogel
Athletes	High VO_2_ Max Demand for O_2_	Rapid speed of action Short control time for competition Cannot enhance performance Easy to detect	Inhaled (liposome) Inhaled (nanocarrier) Transdermal
Co-Morbid Ex: COPD	Low inspiratory capacity Low VO_2_ Max Chronic inflammation Frequent exacerbation	Rapid speed of action Long lasting Cross-control of COPD Combination drugs	Inhaled (micellar) Inhaled (nanocarrier) Polymer Diffusion Hydrogel

## Data Availability

Data on reported clinical trials are available from clinicaltrials.gov (accessed on 21 December 2021) or their respective publications. No new, unreported datasets were used.

## Abbreviation

AERD	Aspirin-Exacerbated Respiratory Syndrome
ACOS	Asthma-COPD Overlap Syndrome
AG	Andrographolide
ATP	Adenosine Triphosphate
BALB/C	Bagg and Albino Mice (White Lab Mice)
COPD	Chronic Obstructive Pulmonary Disorder
DMPC	Dimyristoyl-sn-glycero-3-phosphocholine
DPI	Dry Powder Inhaler
FACET	Formoterol and Corticosteroids Establishing Therapy
FEV1	Forced Expiratory Volume in the 1st Second
GERD	GastroEsophageal Reflux Disease
GOAL	Gaining Optimal Asthma controL
HSPC	Hydrogenated Soybean Phosphatidylcholine
hUCMSCs	Human Umbilical Cord MSCs
ICS	Inhaled Corticosteroids
IL	Interleukin
ILC2s	Group 2 Innate Lymphoid Cells
LABA/LAMA	Long-Acting Beta/Muscarinic Agonist
LRA	Leukotriene Receptor Antagonist
miR-1470	Micro RNA 1470
MSC	Mesenchymal Stem Cell
MSC-Exo	Exosomes Derived from Mesenchymal Stem Cells
PLGA	Polylactide-co-glycolide
pMDI	Pressurized Metered Dose Inhaler
poly(I:C)	Polyinosin-polycytidylic acid
sEV	MSC-small Extracellular Vesicles
SPIO	Superparamagnetic Nanoparticles of Iron Oxides
